# Urinary Cell Adhesion Molecule 1 Is a Novel Biomarker That Links Tubulointerstitial Damage to Glomerular Filtration Rates in Chronic Kidney Disease

**DOI:** 10.3389/fcell.2019.00111

**Published:** 2019-06-27

**Authors:** Man Hagiyama, Yoshihisa Nakatani, Yasutoshi Takashima, Takashi Kato, Takao Inoue, Ryuichiro Kimura, Tomoyuki Otani, Yasufumi Sato, Hideo Mori, Shuji Arima, Akihiko Ito

**Affiliations:** ^1^Department of Pathology, Faculty of Medicine, Kindai University, Osakasayama, Japan; ^2^Division of Nephrology, Department of Internal Medicine, Faculty of Medicine, Kindai University, Osakasayama, Japan; ^3^Department of Pathology, Osaka Rosai Hospital, Sakai, Japan

**Keywords:** chronic kidney disease, tubulointerstitial lesion, cell adhesion molecule 1, ectodomain shedding, ELISA

## Abstract

Cell adhesion molecule 1 (CADM1) is an immunoglobulin superfamily member strongly expressed on renal tubular epithelia in the urinary tract. Enzymatic cleavage of its ectodomain increases in chronic kidney disease (CKD), and is assumed to contribute to tubulointerstitial lesion formation. Because the cleaved ectodomain fragments are likely to be released into the urine, a sandwich enzyme-linked immunosorbent assay (ELISA) system for urinary CADM1 was developed using two anti-ectodomain antibodies. Urinary CADM1 concentrations in patients with CKD based on various forms of glomerulonephritis and nephropathy (*n* = 127) were measured. A total of 44 patients (35%) had elevated CADM1 concentrations over the normal upper limit (362 pg/mL), with a mean of 1,727 pg/mL. Renal biopsy specimens of all patients were pathologically scored for tubulointerstitial lesions using epithelial degeneration, interstitial inflammation, and fibrosis. There were no correlations between urinary CADM1 concentrations and pathological scores or any widely used renal markers, including glomerular filtration rate (GFR), but there was a weak inverse correlation between pathological scores and GFR (*R^2^* = 0.292). Notably, this correlation gradually increased in patients with increasing CADM1 concentrations, and reached a maximum *R*^2^ (0.899) at a cutoff of 1,569 pg/mL. The results of this study suggest that urinary CADM1 is a useful marker indicating tubulointerstitial damage from elevated GFR levels in CKD.

## Introduction

Chronic kidney disease (CKD) is defined as a condition involving gradual loss of renal function over a period of months or years, and includes diverse diseases that are primarily inflammatory, immunological, metabolic, and circulatory (Webster et al., 2017). Although CKD may start in the glomeruli, tubules, or interstitium, the cortical lesions are usually steadily progressive and evolve into patterns with shared histopathological characteristics of glomerulosclerosis, tubular degeneration, interstitial inflammation, and fibrosis (Satirapoj et al., 2012). Once CKD reaches this stage, the glomerular filtration rate (GFR) and tubulointerstitial damage negatively affect each other via proteinuria and tubuloglomerular feedback (Nangaku, 2004). Many past studies have shown that tubulointerstitial damage correlates with progressive decline in renal function (Risdon et al., 1968; Schainuck et al., 1970; Mackensen-Haen et al., 1981; Nath, 1992; D’Amico et al., 1995). Several tubular biomarkers, including β2-microglobulin (β2MG) and *N*-acetyl-β-D-glucosaminidase (NAG), have long been used to indicate tubular damage, but new biomarkers, such as liver-type fatty acid-binding protein and kidney injury molecule 1, have recently been established as more sensitive indicators (Piscator, 1991; Fiseha and Tamir, 2016; Gluhovschi et al., 2016). In clinical practice, patients with CKD often must undergo renal biopsy to monitor the severity of tubulointerstitial damage as well as to facilitate a diagnostic pathological classification (Hsu et al., 2017). These protocols have been used because the marker molecules are probably released from damaged tubular cells into the urine, and are sensitive to subtle damages in tubular cells. However, they are not sufficient to reflect the entire scope of tubulointerstitial damages, including interstitial inflammation and fibrosis.

We recently described a new mechanism to assess tubulointerstitial damage during CKD (Kato et al., 2018). Cell adhesion molecule 1 (CADM1) is a member of the immunoglobulin superfamily that is expressed in renal tubular cells (Nagata et al., 2012), and is enzymatically shed in the juxtamembrane region of the extracellular domain (Nagara et al., 2012; Mimae et al., 2014). In diabetic nephropathy and arterionephrosclerosis, this ectodomain shedding is increased, and causes tubular epithelial apoptosis (Kato et al., 2018). When we evaluated tubulointerstitial lesions in terms of the three pathological categories of inflammation, epithelial degeneration, and interstitial fibrosis, we found a positive correlation between the degree of total tubulointerstitial damage and the rate of CADM1 ectodomain shedding in the kidney (Kato et al., 2018). Increased CADM1 shedding appeared to cause tubular cell apoptosis, and was involved in interstitial inflammation and fibrosis. CADM1 shedding produces two molecular fragments: the C-terminal remnant on the cell membrane (αCTF) and the N-terminal fragment (NTF) separated from the cell surface (Nagara et al., 2012; Mimae et al., 2014). The NTF is likely released into the tubular lumen, i.e., into the urine. If this occurs, the urinary NTF concentration increases during tubulointerstitial damage, and the damage is exacerbated. In our screening of CADM1 tissue distributions, we found that CADM1 expression was restricted to renal tubular cells lining the epithelia of the urinary tract lumens and cavities. We therefore proposed that urinary CADM1 is a good biomarker to estimate the degree of tubulointerstitial damage during CKD.

In the present study, we developed a sandwich enzyme-linked immunosorbent assay (ELISA) for urinary CADM1 using two monoclonal antibodies against the ectodomain of CADM1, to measure CADM1 concentrations in the urine of CKD patients undergoing renal biopsy. We compared the CADM1 concentrations with pathological scores of the tubulointerstitial lesions of biopsy specimens and with conventional renal biomarkers. We also conducted Western blot analyses of the urine samples to confirm that the urinary CADM1 measured by the ELISA was CADM1-NTF, and used cell culture experiments to determine whether CADM1 shedding was induced in tubular cells by ischemia.

## Materials and Methods

### Cells, Proteins, and Antibodies

A mouse L fibroblast subclone that exogenously expressed full-length CADM1 was established previously (Koma et al., 2004). Rabbit CNT renal distal tubular cells were described previously (Takahashi and Suzuki, 1994). A recombinant soluble isoform of CADM1 fused with the Fc portion of human IgG1 (sCADM1-Fc) and the deletion form that lost the three Ig-like loops from sCADM1-Fc (ΔsCADM1-Fc) were purified as described previously (Koma et al., 2004; Hagiyama et al., 2009), and the concentrations were determined using the Bradford assay (Bio-Rad Laboratories, Hercules, CA, United States). Antibodies against the CADM1 ectodomain (3E1 and 9D2, chicken monoclonal) and the C-terminus (rabbit polyclonal) were described previously (Furuno et al., 2005; Hagiyama et al., 2009). 3E1 and 9D2 were biotinylated using the Biotin Labeling Kit-NH_2_ (Dojindo Molecular Technologies, Inc., Kumamoto, Japan) according to the manufacturer’s instructions. Other primary antibodies used in this study targeted human IgG Fc (goat polyclonal; Jackson ImmunoResearch Laboratories, West Grove, PA, United States) and β-actin (Medical & Biological Laboratories, Nagoya, Japan). Peroxidase-conjugated secondary antibodies were purchased from Amersham (Buckinghamshire, United Kingdom). A chicken IgY clone (U04) was purchased from R&D Systems (Minneapolis, MN, United States).

### Sandwich ELISA

The 96-well microtiter plates were coated with 100 μL/well phosphate-buffered saline (PBS) containing 9D2 monoclonal antibodies (0.1 μg) and allowed to adhere overnight at 4°C. The plates were washed with Tris-buffered saline supplemented with Tween 20 (0.05%) (TBS-T) and blocked with 300 μL/well of 5% (w/v) bovine serum albumin (BSA) in PBS for 1 h at room temperature. Following five washes with TBS-T, 100 μL aliquots of test samples or sCADM1-Fc as a standard that was serially diluted (40 ng/mL to 1.28 pg/mL) in PBS containing 1% BSA, were added in triplicate and duplicate, respectively, to the wells and incubated at room temperature for 2 h. After five washes with TBS-T, 100 μL of biotinylated 3E1 monoclonal antibody (0.3 ng/mL) was added to each well and incubated for 1 h at room temperature. In parallel with this incubation, avidin–biotin complexes were formed by mixing 1 μL Reagent A (avidin DH) with 1 μL Reagent B (biotinylated alkaline phosphatase) from the Vectastain ABC-AP Staining Kit (Vector Laboratories, Burlingame, CA, United States) in 100 μL TBS-T for 30 min at room temperature, then diluted 100× in TBS-T immediately before use. The wells were washed five times with TBS-T, and then 100 μL of freshly prepared Reagents A and B mixture solution was added to each well and incubated for 1 h at room temperature. During this incubation, a solution containing CSPD, an alkaline phosphatase substrate, was prepared using the BM Chemiluminescence ELISA Substrate (AP) (Sigma–Aldrich, St. Louis, MO, United States) according to the manufacturer’s instructions. The wells were washed five times with TBS-T and four times with Tris-buffered saline, and then 100 μL of the freshly prepared CSPD solution was added to each well and incubated for 45 min at room temperature. The chemiluminescence from each well was measured using a plate reader (Wallac 1420 Arvo Mx2 luminometer; PerkinElmer, Boston, MA, United States).

Validation of the developed ELISA was conducted essentially according to the guidelines of the International Organization of Standardization and the National Committee for Clinical Laboratory Standards Evaluation protocols (International Organization for Standardization, 2008). Recombinant sCADM1-Fc protein was used as a quality control (QC). Intra-assay precision was determined by four repeated measurements of each QC sample in a plate, and inter-assay precision was established by assessing each QC sample across three different plates with quadruple wells.

### Human Samples

The present study enrolled all patients who underwent renal biopsy at Kindai University Hospital (Osaka, Japan) between July 2015 and March 2018. Some patients were excluded because they suffered from nephritis related to a particular medication, such as anti-cancer drugs or immunosuppressants, or experienced acute inflammatory symptoms such as glomerulonephritis with crescent formation. Urine samples were collected from the patients ≤ 30 min before they underwent renal biopsy, and were stored at 4°C for <2 weeks until they were analyzed using ELISA. Renal biopsy specimens were fixed in 10% phosphate-buffered formalin immediately after biopsy, embedded in paraffin, cut into sections, and stained with several dyes and methods including hematoxylin and eosin, periodic acid Schiff, periodic acid methenamine silver, and Masson’s trichrome. Pathological diagnosis was determined independently by at least two senior pathologists, and was confirmed by senior nephrologists (YN and SA). The healthy volunteers consisted of 15 males and eight females, with ages ranging from 18 to 45 (mean 28) years. All experiments were approved by the ethics committee of Kindai University Faculty of Medicine (No. 26-279). This study was conducted in accordance with the tenets of the Declaration of Helsinki and its subsequent amendments. Written informed consent was obtained from all patients.

Other materials and methods are described in the Supplementary Methods.

## Results

### Development of a Sandwich ELISA for Quantification of CADM1 in Urine

**FIGURE 1:**
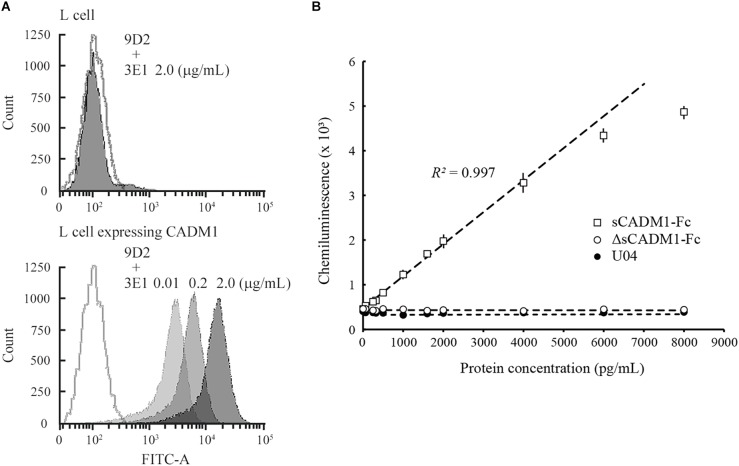
Development of a sandwich ELISA for cell adhesion molecule 1 (CADM1). **(A)** Fluorescence-activated cell sorting (FACS) analysis of L cells expressing full-length CADM1 using 3E1 and 9D2 antibodies. Original L (upper) or CADM1-expressing L cells (lower) were incubated serially, first with 9D2 and then with 3E1 (0.01, 0.2, or 2 μg/mL; bright to dark gray-painted), or incubated in buffer without any primary antibodies (gray-lined, open), and were then analyzed using FACS. **(B)** Calibration curve of the CADM1 enzyme-linked immunosorbent assay (ELISA) for sCADM1-Fc. The ELISA was developed using 9D2 and biotinylated 3E1 antibodies as capture and detection antibodies, respectively. Chemiluminescence intensities were measured for recombinant proteins sCADM1-Fc and ΔsCADM1-Fc, and U04 chicken IgY using the ELISA, were shown as dots with bars indicating the SDs, and were then plotted with concentrations determined using the Bradford assay and serially diluted. Note that most of the SDs are too small to be shown in the graph. Representative results are displayed, with the approximate line and curve (dotted lines). Pearson’s *R*^2^ corresponding to the linear approximation for sCADM1-Fc is shown (range: 0–4,000 pg/mL).

Mouse L fibroblasts and subcloned cells that expressed the full-length form of CADM1 were incubated serially with two anti-CADM1 ectodomain antibodies, first 9D2 and then 3E1, and were subjected to fluorescence-activated cell sorting (FACS) analyses. L cells did not react with either antibody, whereas CADM1-expressing L cells reacted with both antibodies (Figure 1A). The fluorescence intensities bound to the subclone increased as the 3E1 concentration increased, indicating that 9D2 and 3E1 recognized different epitopes in the CADM1 ectodomain (Figure 1A).

To develop a sandwich ELISA for urinary CADM1, we biotinylated one of the two antibodies to use as a detection antibody and used the other antibody as the capture antibody. We examined which antibody was suitable as the detection antibody using a recombinant soluble isoform of CADM1 fused with the Fc portion of human IgG1 (sCADM1-Fc) (Koma et al., 2004), and optimized both the quantity of the capture antibody to be coated on the plate and the dilution factor for detecting the antibody. After multiple trials, we decided to use 0.1 μg of 9D2 to coat each well as the capture antibody, combined with 0.3 ng/mL biotinylated 3E1 as the detection antibody. The color (chemiluminescence) was developed using alkaline phosphatase-conjugated avidin and the chemiluminescent substrate for alkaline phosphatase. There was no cross-reactivity against the deletion form of sCADM1-Fc that lost the three Ig-like loops from sCADM1-Fc (ΔsCADM1-Fc) (Hagiyama et al., 2009) or U04, an IgY clone unrelated to CADM1 (Figure 1B).

A representative standard dose–response curve for sCADM1-Fc is shown in Figure 1B. The limit of detection (LOD) and lower limit of quantification (LLOQ) were determined to be 60 pg/mL and 213 ng/mL, respectively, as calculated by the formulas: LOD = average luminescence of the blank + 3 × [SD of the blank], and LLOQ = average luminescence of the blank + 10 × (SD of the blank) (Currie, 1968; Findlay et al., 2000; Dixit et al., 2010). In particular, for each assay, the SD was calculated from the measured values of two blank wells that were prepared for standard dose–response curve drawing, and then the averages of 3 × SD and 10 × SD were calculated from 24 assays and were regarded as LOD and LLOQ, respectively. According to the standard dose–response curve, the linear quantification range was approximately 0–4,000 pg/mL, with an excellent linearity between luminescence and sCADM1-Fc concentration (*R*^2^ > 0.99) (Figure 1B). The upper limit of quantification (ULOQ) was set at 4,000 pg/mL. When the urine samples contained CADM1 concentrations over the ULOQ, they were diluted with PBS and measured in the linear range.

The assay precision was validated using sCADM1-Fc spiked in PBS at three different doses of high, middle, and low concentrations (Samples A, B, and C, respectively; Table 1). We measured these spiked samples four times in the same assay at different time points to determine the coefficient of variation (CV) for intra-assay precision, and also measured these samples once a day for 3 consecutive days to determine the CV for inter-assay precision. The CV for each was less than 10%, indicating that the developed ELISA system had good precision (Table 1).

**Table 1:** Validation of the CADM1 ELISA kit.

Sample		Intra-assay precision		Inter-assay precision	
		
	Spiked conc. (pg/mL)	Measured conc. (pg/mL)	CV (%)	Measured conc. (pg/mL)	CV (%)
A	3,000	3,077 ± 108	1.4 ± 0.7	3,080 ± 20	3.3 ± 0.8
B	1,500	1,476 ± 65	2.7 ± 2.1	1,485 ± 25	3.6 ± 1.4
C	300	306 ± 11	5.5 ± 2.9	303 ± 15	4.5 ± 4.0


*For all measurements, the samples were prepared in triplicate. Measured concentrations (conc.) and coefficients of variation (CV) are expressed as the mean ± SD calculated from three independent intra-assays and inter-assays. CADM1, cell adhesion molecule 1; ELISA, enzyme-linked immunosorbent assay.*

### Measurement of Urinary CADM1 Concentrations Using the Sandwich ELISA

Urine samples from healthy volunteers (*n* = 23) were examined using the developed ELISA. Urinary CADM1 concentrations of 16 volunteers were lower than the LLOQ (213 pg/mL), and those of the remaining volunteers had a mean of 269 pg/mL. The upper limit of the normal CADM1 concentration was estimated as 362 pg/mL, the value of the mean (161 pg/mL) + [2.58 × SD (78 pg/mL)], by tentatively allocating the half-value of LLOQ (107 pg/mL) to the former 16 volunteers to avoid overestimating or underestimating the data variance (Hashimoto et al., 2015).

**FIGURE 2:**
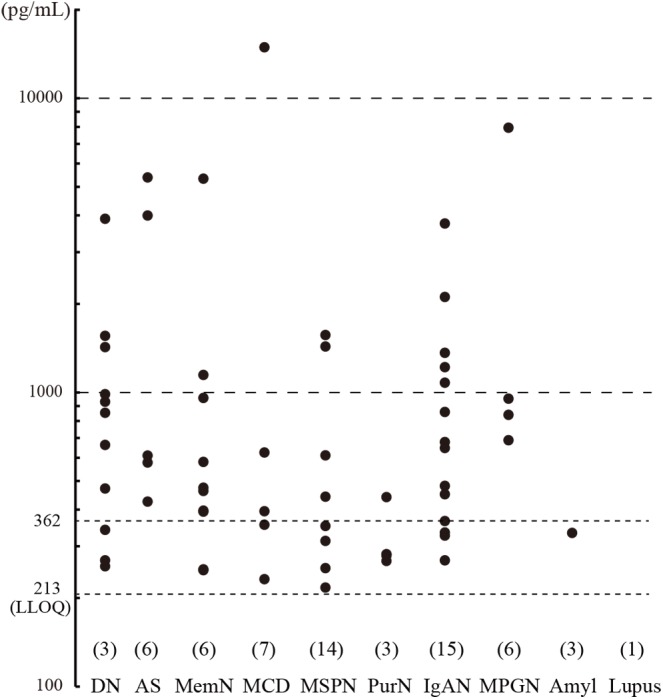
Urinary cell adhesion molecule 1 (CADM1) concentrations in patients with chronic kidney disease (CKD). Urine samples were collected from CKD patients who underwent renal biopsy, and their CADM1 concentrations were measured using ELISA. Patients were classified into 10 groups according to primary diseases. The parentheses immediately above the disease names show the numbers of patients whose CADM1 concentrations were below the lower limit of quantification (LLOQ; 213 pg/mL). The *Y*-axis is logarithmic. DN, diabetic nephropathy; AS, arterionephrosclerosis; MemN, membranous nephropathy; MCD, minor change disease; MSGN, mesangioproliferative glomerulonephritis; PrurN, pruritic nephritis; IgAN, IgA nephropathy; MPGN, membranoproliferative glomerulonephritis; Amyl, amyloid nephropathy; Lupus, lupus nephritis.

Urine samples were collected from CKD patients immediately before undergoing renal biopsy at Kindai University Hospital (*n* = 127), and urinary CADM1 concentrations were measured using ELISA. Characteristics of the patients are summarized in Table 2. Urinary CADM1 concentrations over the upper normal limit (362 pg/mL) were detected in 44 patients (35%), and ranged up to 14,899 pg/mL, with a mean of 1,727 pg/mL (Figure 2). A total of 64 patients (50%) had urinary CADM1 concentrations below the LLOQ (213 pg/mL); the half-value of LLOQ (107 pg/mL) was tentatively allocated to these patients in the present statistical analyses (Figure 2). The patients were classified into 10 groups according to the primary disease, which was diagnosed histologically using the individual biopsy specimens. The diseases included various forms of glomerulonephritis and nephropathy that were generally recognized to cause CKD (Table 2). There was at least one patient with an increased urinary CADM1 concentration (>362 pg/mL) in every group of diseases except for the smallest two groups, amyloid nephropathy (*n* = 4) and lupus nephropathy (*n* = 1) (Figure 2). CADM1 concentrations were not associated with the pathological classification of primary diseases (minimal *P* = 0.36 for diabetic nephropathy vs. mesangioproliferative glomerulonephritis).

**Table 2:** Patient characteristics.

	*n*			
		
Renal disease		Sex		Age range (median)
			
		M	F	
Diabetic nephropathy	14	10	4	40–84 (64)
Arterionephrosclerosis	11	7	4	28–82 (75)
Membranous nephropathy	16	9	7	42–81 (71)
Minor change disease	12	4	8	36–80 (67)
Mesangioproliferative glomerulonephritis	22	13	9	20–79 (56)
Pruritic nephritis	8	4	4	31–85 (67)
IgA nephropathy	29	14	15	19–79 (43)
Membranoproliferative glomerulonephritis	10	6	4	23–85 (74)
Amyloid nephropathy	4	1	3	57–76 (70)
Lupus nephritis	1	1	0	28
Total	127	69	58	19–85 (63)


*M, male; F, female.*

**FIGURE 3:**
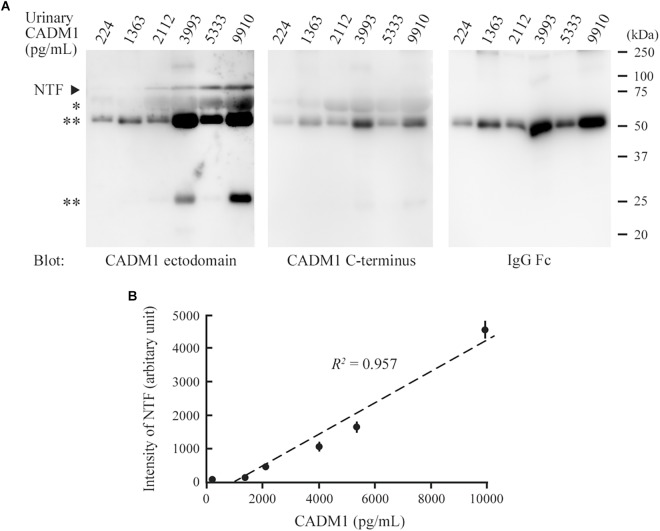
Comparison between Western blot analysis and the enzyme-linked immunosorbent assay (ELISA) for the detection of urinary cell adhesion molecule 1 (CADM1). **(A)** Western blot analyses of urine samples for CADM1. Urine samples with various CADM1 concentrations (measured by ELISA) were blotted in triplicate with antibodies against the ectodomain (3E1) or C-terminus of CADM1. The other blot was probed with an anti-human IgG Fc antibody to detect IgG in urine. ^∗^ and ^∗∗^ indicate albumin and IgG (heavy and light chains), respectively, that were detected non-specifically in the blots for CADM1. **(B)** Scatter plots of urinary CADM1 concentrations (ELISA) and the band intensities of NTF detected by 3E1 (Western blot analyses). The means of the N-terminal fragment intensities from three independent Western blot analyses are plotted as dots with bars indicating the SDs, and are approximated (dotted line). Pearson’s *R*^2^ is shown.

We conducted Western blot analyses on several urine samples that showed CADM1 at low, middle, or high concentrations using ELISA. CADM1-NTF was detected on the blot using 3E1, and its densitometric intensity was well correlated with the urinary CADM1 concentrations measured by ELISA (*R*^2^ = 0.957; Figure 3A,B). However, the antibody against the CADM1 C-terminus did not yield specific immunoreactive bands in any samples, although both CADM1 antibodies appeared to cross-react with urinary IgG (Figure 3A). Urinary CADM1 detected by the ELISA therefore appeared to be exclusively CADM1-NTF. Note that because sCADM1-Fc used as the standard protein has a somewhat larger molecular weight than CADM1-NTF, the measured values of urinary CADM1 concentrations in this study are useful just as the values relative to each other, and should be corrected accordingly in future studies.

### The Correlations of Urinary CADM1 With Tubulointerstitial Damage and Renal Biomarkers

**FIGURE 4:**
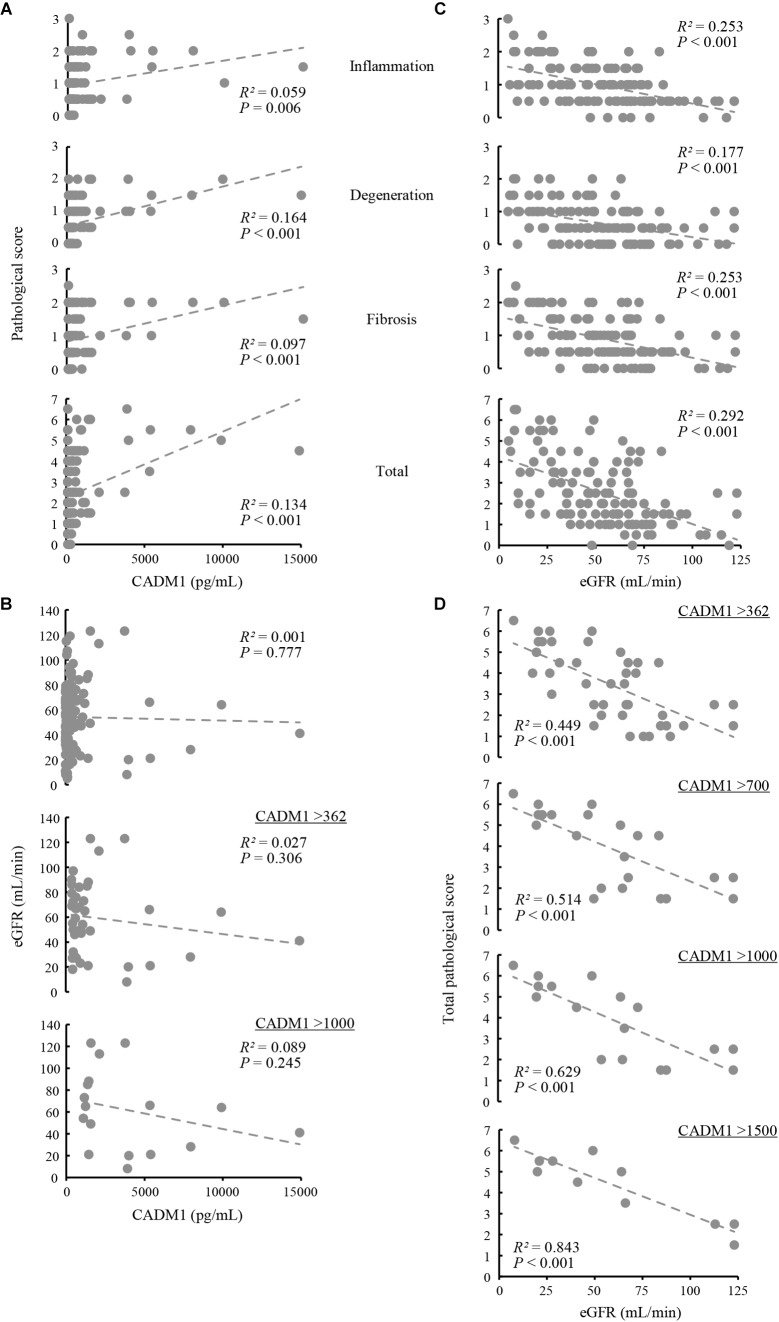
Correlation among urinary cell adhesion molecule 1 (CADM1) concentrations, pathological scores, and the estimated glomerular filtration rate (eGFR). **(A)** Urinary CADM1 concentration (pg/mL) vs. pathological scores (inflammation, degeneration, fibrosis, and total). **(B)** Urinary CADM1 concentration (pg/mL) vs. eGFR (mL/min). **(C,D)** eGFR (mL/min) vs. pathological scores (inflammation, degeneration, fibrosis, and total) in a group including all patients **(C)**. In **D**, urinary CADM1 concentrations (pg/mL) were used as a cutoff to select patients, as indicated. Approximate lines (dotted line), Pearson’s *R*^2^, and *P*-values are shown.

Renal biopsy sections were pathologically evaluated, and tubulointerstitial damage was rated on a scale of 0–3 in each of three aspects of tubular epithelial degeneration involving interstitial inflammation, lymphocyte infiltration, and fibrosis. The three scores were summed for each patient and expressed as the total pathological score, which indicated the degree of overall damage of the tubulointerstitial lesions. Correlations between urinary CADM1 concentrations and these four pathological scores were analyzed. In an analysis of the entire patient cohort, CADM1 concentration was not correlated with any of the four scores, or with the estimated GFR (eGFR) (Figure 4A,B). The same result was found in an analysis of a selected group of patients who had elevated CADM1 concentrations (over the normal upper limit of 362 pg/mL) (Figure 4B and Supplementary Figure S1).

We next analyzed whether the pathological scores correlated with the eGFR. A weak inverse correlation was found between the eGFR and the total pathological score in a group including all patients (*R*^2^ = 0.292), as well as between the eGFR and inflammation or fibrosis scores (*R*^2^ = 0.253 for each) (Figure 4C). This correlation was stronger in a selected group of patients with higher CADM1 concentrations (>362 pg/mL; *R*^2^ = 0.449) (Figure 4D). When patients with higher CADM1 concentrations, ranging from 700 and 1,000 to 1,500 pg/mL were selected, the correlation gradually became stronger, with *R*^2^ values reaching 0.84 (Figure 4D). Notably, the approximated lines were nearly identical among the three scatter plots of patient groups selected by CADM1 concentrations of >700, 1,000, and 1,500 pg/mL (Figure 4D). The optimal CADM1 concentration cutoff that maximized the *R*^2^ value (0.899) was calculated as 1,569 pg/mL, with a *P*-value of 3.02E-05.

Urinary CADM1 concentrations were then compared with the conventional urinary tubular biomarkers β2MG and NAG, but no significant correlation was found with either of the two markers (Supplementary Figure S2A). The correlation between eGFR and the tubulointerstitial pathological score was not significant when the patients were selected using high values of either β2MG or NAG as a cutoff (Supplementary Figure S2B). In the present group of patients, there was no correlation between β2MG and NAG (Supplementary Figure S2C).

### Induction of CADM1 Shedding and Apoptosis in CNT Renal Tubular Cells Under Ischemia-Like Conditions

**FIGURE 5:**
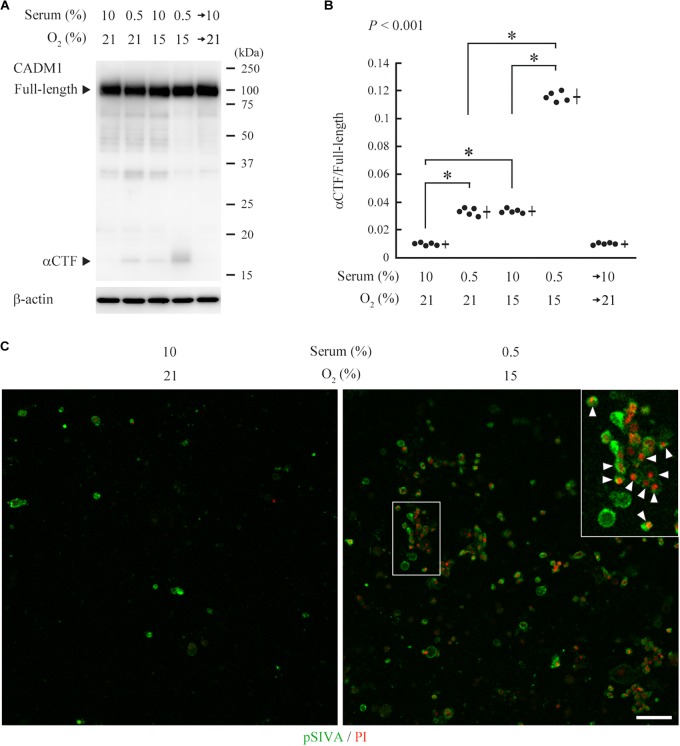
Induction of urinary cell adhesion molecule 1 (CADM1) ectodomain shedding and apoptosis in CNT renal tubular cells under ischemia-like conditions. **(A)** CNT cells were cultured for 3 days under normal conditions [10% fetal calf serum and 21% O_2_] or ischemia-like hypoxic conditions [10% serum and 15% O_2_, serum-starved (0.5% serum and 21% O_2_)], or both (0.5% serum and 15% O_2_). The combined condition was restored to normal, and the cell culture was continued for another 2 days (serum→10% and O_2_→21%). Cell lysates were analyzed by Western blotting with anti-CADM1 C-terminus antibody (upper panel). The blot was reprobed with an anti-β-actin antibody to indicate the amount of protein loading per lane (lower panel). Representative results are shown. **(B)** Expression levels were densitometrically measured from five independent experiments and were plotted. The mean ratios of αCTF/full-length CADM1 and the SDs are depicted as cross lines. *P*-value using one-way ANOVA is shown, and the Bonferroni correction was applied to particular two groups: ^∗^*P* < 0.01. **(C)** CNT cells were cultured for 3 days under normal or ischemia-like conditions as indicated, and were then labeled with pSIVA (green) and propidium iodide (PI; red). A boxed area is enlarged in the inset. Arrowheads indicate pSIVA and PI double-positive cells. Bar = 50 μm.

CNT renal distal tubular cells (Takahashi and Suzuki, 1994) abundantly expressed full-length CADM1 without accompanying αCTF under standard culture conditions (10% serum and 21% O_2_), indicating that CADM1 shedding did not occur (Figure 5A). CNT cells were cultured for 3 days under conditions mimicking *in vivo* ischemia, i.e., serum-starvation (0.5% serum), hypoxia (15% O_2_), or both. The expression of αCTF was detectable in association with a reduction in the full-length level, and these changes in CADM1 concentrations were increased under combined conditions (0.5% serum and 15% O_2_), suggesting that ischemia caused CADM1 shedding in distal tubules (Figure 5A,B). When the ischemia-like conditions were restored to standard conditions, CADM1 expression returned to its original level in 2 days (Figure 5A,B).

pSIVA is an annexin-based fluorescent biosensor that binds to phosphatidylserine exposed on the outer leaflet of the cell membrane. It can be monitored in living cells during the progression of the apoptotic pathway from early stages to complete cell death (Kim et al., 2010). We examined whether the induced CADM1 shedding is associated with apoptotic reactions in CNT cells, using a combination of pSIVA and propidium iodide (PI), a fluorescent indicator for late-stage cell death. We observed a few pSIVA- or PI-positive CNT cells under standard culture conditions (Figure 5C and Supplementary Figure S3). In contrast, when cells were cultured under ischemia-like conditions for 3 days, the number of pSIVA- and PI-positive cells increased, and some cells were double-positive (Figure 5C).

## Discussion

We have developed a sandwich ELISA for urinary CADM1 using two monoclonal antibodies against the ectodomain of CADM1. The normal concentration of urinary CADM1 was below the LLOQ (213 pg/mL), and its upper limit was estimated to be 362 pg/mL. The ELISA was therefore sensitive enough to detect an abnormal increase in urinary CADM1 concentration. Overall, our ELISA identified increased CADM1 concentrations in 39% of patients examined. Western blot analyses of urine samples from these patients confirmed that the form of urinary CADM1 detected by the ELISA was exclusively CADM1-NTF, consistent with our hypothesis that increased CADM1 shedding in tubular epithelial cells was involved in the pathogenesis of CKD (Kato et al., 2018).

Urinary CADM1 concentrations were not associated with primary renal diseases, tubulointerstitial pathological scores, eGFR, β2MG, or NAG, but were useful for selecting patients with strong correlations between tubulointerstitial damage and eGFR. Although the precise mechanism underlying this correlation remains unknown, this function of CADM1 may be based on the finding that CADM1 is expressed in the distal tubule (Nagata et al., 2012), unlike β2MG or NAG, which are derived from the proximal tubule (Bazzi et al., 2002; Zeng et al., 2014). It is known that proximal tubules are sensitive to ischemia, whereas distal tubules are resistant (Jennette et al., 2015; Chevalier, 2016). Urinary CADM1 may subsequently increase when ischemia is relatively severe in the renal cortex. Consistent with this possibility, CADM1 shedding was induced in CNT distal tubular cells under ischemia-like conditions, in association with the induction of cell apoptosis (Figure 5). In addition, distal tubules contain the macula densa, the epithelial cellular region adjacent to the glomerulus and responsible for tubuloglomerular feedback (Ren et al., 2004; Ferenbach and Bonventre, 2016). Patients with increased urinary CADM1 may have cellular degeneration in the macula densa and therefore tend to develop defective tubuloglomerular feedback. Because CADM1 is derived from the distal tubule, urinary levels of CADM1 may reflect tubulointerstitial lesions that are severe enough to reduce the GFR.

However, increased urinary CADM1 levels were not a direct hallmark of severe tubulointerstitial damage, but instead may have indicated that the severity was strongly correlated with eGFR. As shown in Figure 4D, there were some patients with elevated urinary CADM1 concentrations who also had good eGFR and low pathological scores. Because urinary CADM1 reflects cortical ischemia, as discussed in the previous paragraph, the prognoses of these patients may be defined by the concurrence of a future low GFR and severe tubulointerstitial damage. For these patients, the time point of CADM1 measurement in the present study may simply have been in the very early stage of their disease progression. We are planning follow-up studies to examine this possibility.

The mechanisms involved in increasing CADM1 levels remain to be fully elucidated. Notably, ischemia has been reported to upregulate ADAM10, a candidate protease responsible for CADM1 shedding in distal tubules (Schramme et al., 2008; Herzog et al., 2014). When considering that urinary CADM1 concentrations were not correlated with the pathological classification of renal disease, patients with elevated urinary CADM1 may have originally had (or been genetically predisposed to) anatomical or functional defects in the cortical vasculature that made them susceptible to developing local ischemia. Previous studies have suggested that cortical ischemia may be a central factor in the progressive nature of CKD (Shoji et al., 2014; Fu et al., 2016), and the results of the present study are consistent with this possibility.

Based on the urinary CADM1 concentrations found in the present study, it may be clinically meaningful to group CKD patients by their urinary CADM1 concentrations, independently of histological typing. The percentages of patients with CADM1 concentrations > 362, > 1000, and > 1,500 pg/mL were 35, 14, and 9%, respectively, and they showed a strong correlation between tubulointerstitial damage and eGFR, with high *R*^2^ values of 0.449, 0.629, and 0.843, respectively. This indicates that in these patients, the degree of tubulointerstitial damage could be estimated from the GFR with an accuracy that increased as the CADM1 concentration increased. These results suggest that urinary CADM1 measurements can potentially replace renal biopsies for monitoring the severity of tubulointerstitial damage in a significant proportion of CKD patients. We are currently designing large cohort studies to verify this clinically valuable possibility.

Western blot analyses of urine samples revealed that urinary CADM1 concentrations reflected the NTF produced by ectodomain shedding that has been postulated to contribute to distal tubular cell apoptosis (Kato et al., 2018). It is reasonable to consider the possibility that NTF released from the tubular epithelia is not only excreted into urine, but is also deposited into the interstitium around the tubules. We previously showed that an interstitial soluble form of CADM1 bound to the full-length CADM1 ectodomain present on cell membranes, which served as a guide molecule for directional outgrowth of nerve fibers (Hagiyama et al., 2009). In addition, the class I-restricted T cell-associated molecule (CRTAM) is known as a *trans*-heterotypic binding partner of CADM1, and is expressed on lymphocytes, including CD8^+^ cytotoxic T cells (Boles et al., 2005; Galibert et al., 2005). Considering that NTF is structurally almost identical to the soluble form of CADM1, NTF accumulated in the renal cortical interstitium is likely to be involved in inflammation by serving as a scaffold for CRTAM-expressing lymphocytes (Arase et al., 2005). This hypothesis merits further testing, as it provides a molecular basis for the general pathological link between epithelial degeneration and concurrent local inflammation (Shanley, 1996; Kumar et al., 2018).

## Conclusion

We developed a sandwich ELISA for urinary CADM1, and showed that urinary CADM1 concentrations increased in a significant proportion of patients with CKD, independent of their primary disease. This new urinary biomarker provides clinically important information, indicating a strong correlation between tubulointerstitial damage and the GFR when CADM1 levels are elevated in CKD. The mechanism underlying this observed correlation is thought to involve the unique distribution of CADM1 in the distal tubule, which is generally known to be resistant to ischemia. Further characterization of this marker will hopefully identify the precise mechanisms underlying the development and progression of tubulointerstitial lesions in CKD patients.

## Data Availability

All datasets generated for this study are included in the manuscript and/or the Supplementary Files.

## Ethics Statement

Human samples: The present study enrolled all patients who underwent renal biopsy at Kindai University Hospital (Osaka, Japan) between July 2015 and March 2018. Some patients were excluded because they suffered from nephritis related to a particular medication, such as anti-cancer drugs or immunosuppressants, or experienced acute inflammatory symptoms such as glomerulonephritis with crescent formation. Urine samples were collected from the patients ≤ 30 min before they underwent renal biopsy, and were stored at 4°C for <2 weeks until they were analyzed using ELISA. Renal biopsy specimens were fixed in 10% phosphate-buffered formalin immediately after biopsy, embedded in paraffin, cut into sections, and stained with several dyes and methods including hematoxylin and eosin, periodic acid Schiff, periodic acid methenamine silver, and Masson’s trichrome. Pathological diagnosis was determined independently by at least two senior pathologists, and was confirmed by senior nephrologists (YN and SA). The healthy volunteers consisted of 15 males and eight females, with ages ranging from 18 to 45 (mean 28) years. All experiments were approved by the ethics committee of Kindai University Faculty of Medicine (No. 26-279). This study was conducted in accordance with the tenets of the Declaration of Helsinki and its subsequent amendments. Written informed consent was obtained from all patients.

## Author Contributions

YN, YT, and SA collected urinary samples and clinical laboratory data from the patients. YT and AI conducted the histological examinations and made pathological diagnoses. MH, YT, and YS developed the ELISA system and measured urinary CADM1 concentrations. MH also conducted cell culture experiments and Western blot analyses. TI, RK, and TO participated in these experiments. HM and MH conducted the statistical analyses. TK and AI conceived and designed the study. AI drafted the manuscript. All authors read and approved the final manuscript.

## Conflict of Interest Statement

YS was employed by the company Epsilon Molecular Engineering, Inc., Saitama, Japan. The remaining authors declare that the research was conducted in the absence of any commercial or financial relationships that could be construed as a potential conflict of interest.
