# Supplementary figures and images for: Urinary Cell Adhesion Molecule 1 Is a Novel Biomarker That Links Tubulointerstitial Damage to Glomerular Filtration Rates in Chronic Kidney Disease
Source: Front Cell Dev Biol. 2019 Jun 27;7:111. doi: 10.3389/fcell.2019.00111 (PMC6610501; doi:10.3389/fcell.2019.00111)

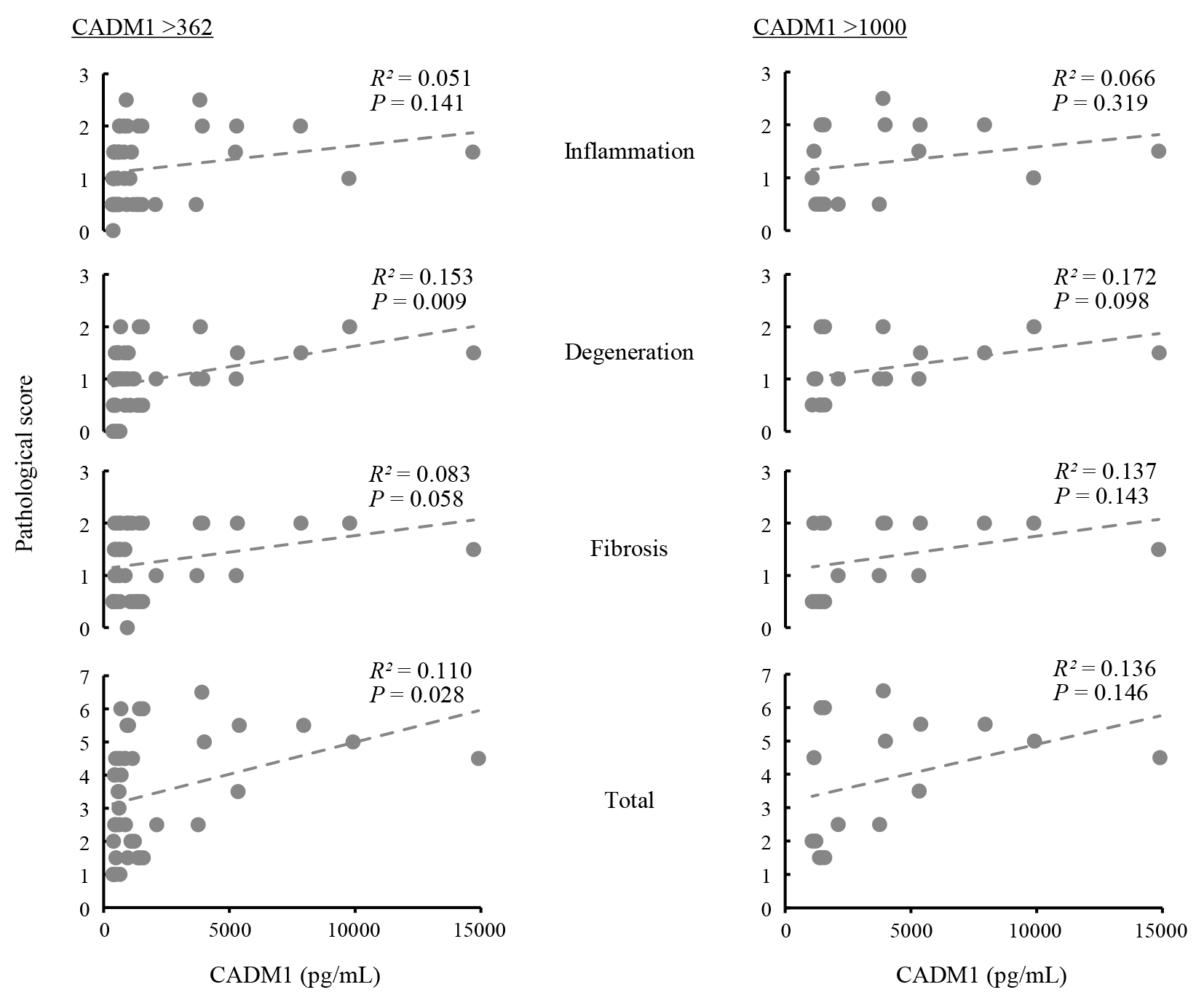

Supplement: Supplementary file 1 [file Image_1.TIF]

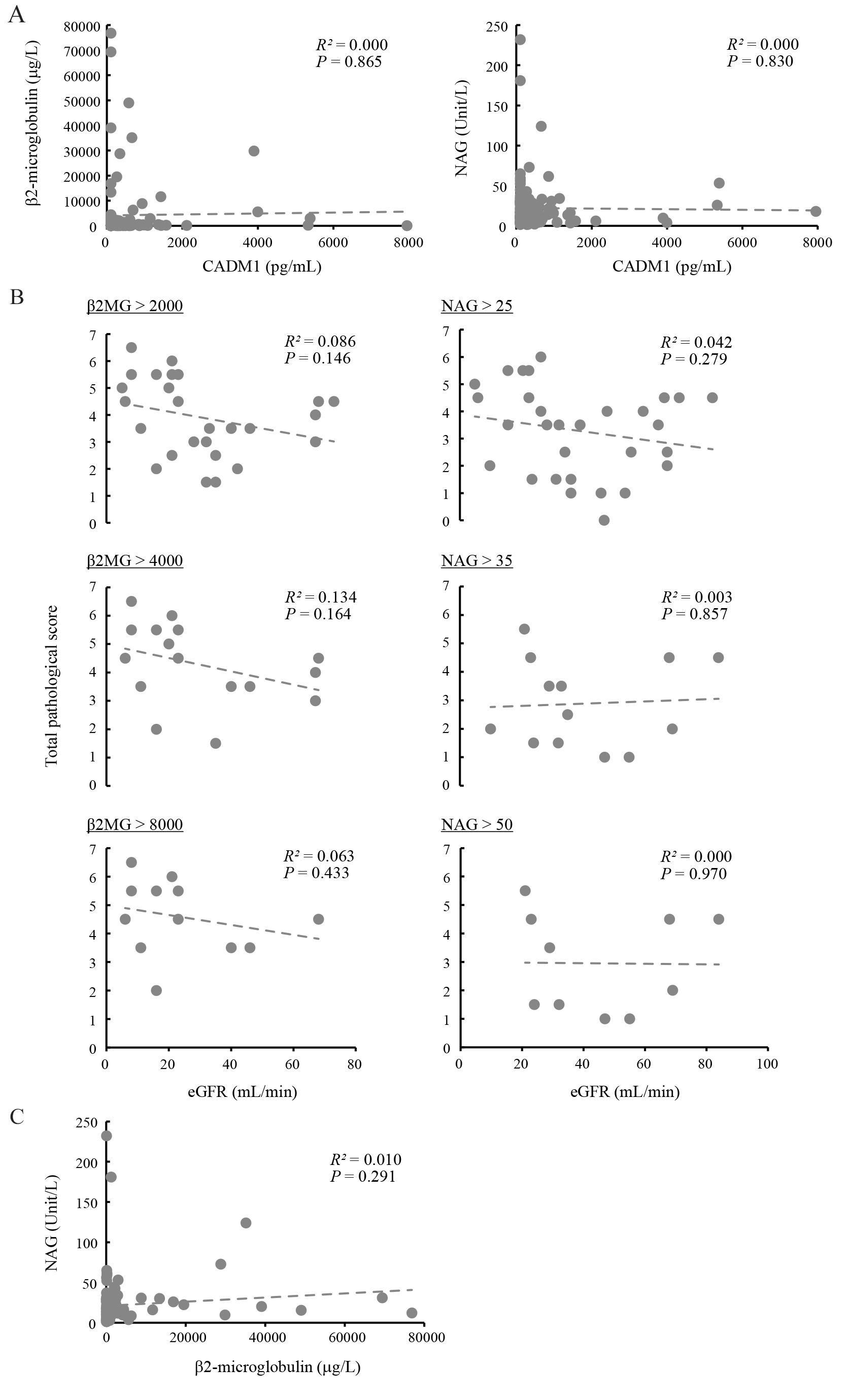

Supplement: Supplementary file 2 [file Image_2.TIF]

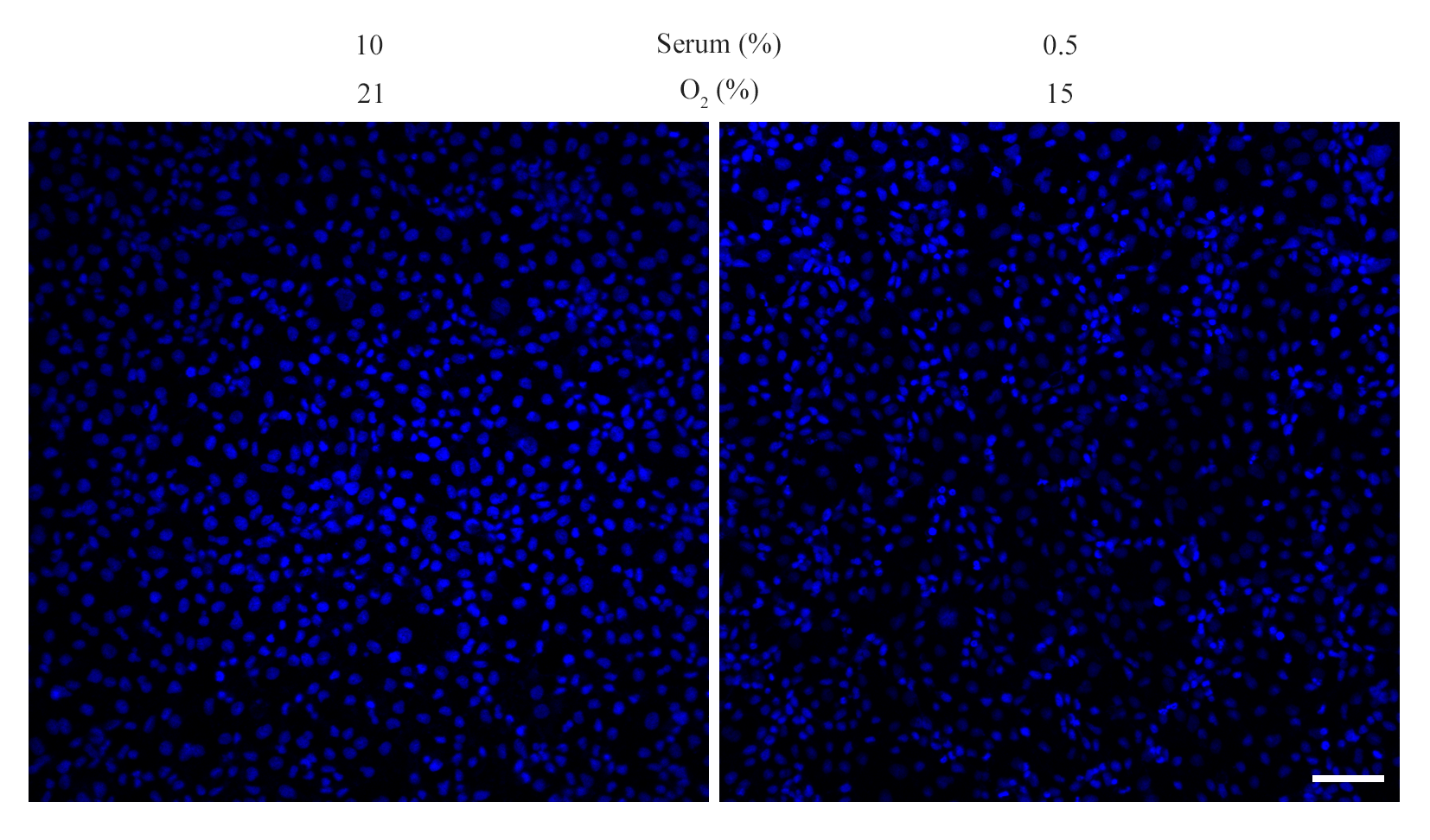

Supplement: Supplementary file 3 [file Image_3.TIF]
