# Supplementary material for: Urinary Cell Adhesion Molecule 1 Is a Novel Biomarker That Links Tubulointerstitial Damage to Glomerular Filtration Rates in Chronic Kidney Disease
Source: Front Cell Dev Biol. 2019 Jun 27;7:111. doi: 10.3389/fcell.2019.00111 (PMC6610501; doi:10.3389/fcell.2019.00111)
Supplement: Supplementary file 4 [file Data_Sheet_1.docx]

**Supplementary material**

**Supplementary Methods**

**FACS analysis**

FACS analysis was performed as described previously (Koma et al., 2004; Furuno et al., 2005). Briefly, L cells were suspended using Accutase Cell Detachment Solution (BD Biosciences, Franklin Lakes, NJ, USA), and harvested by centrifugation, then suspended in cold PBS containing 1% BSA and 0.1% sodium azide (FACS buffer). Cells were incubated serially first with 9D2 (5 μg/mL) and then with 3E1 (0.01, 0.2 or 2 μg/mL), in the buffer for 30 min on ice. After wash with cold FACS buffer, cells were incubated with Alexa Fluor 488-conjugated donkey anti-chicken IgY (Jackson ImmunoResearch Laboratories) at a dilution of 1:500 for 30 min on ice in the dark. After wash with cold buffer, cells were suspended in cold FACS buffer containing PI (20 μg/ml; Sigma-Aldrich, Saint Luis, MO, USA) and RNase A (10 μg/mL; Invitrogen, Carlsbad, CA, USA) and were analyzed with BD FACSCalibur (Becton Dickinson, Franklin Lakes, NJ, USA).

**Pathological scores of tubulointerstitial lesions**

Two pathologists (YT and AI) inspected the pathological specimens, and quantified the severities of tubulointerstitial lesions, as previously reported (Kato et al., 2009; Kato et al., 2018). Briefly, the tubulointerstitial damage was rated on a scale of 0 to 3 in each of three categories of tubular epithelial degeneration, interstitial inflammation (lymphocyte infiltration) and fibrosis. Lesions of each category were graded as follows: 0: no lesion, 1: lesion occupying 25% of the renal cortex, 2: lesion occupying 50% of the cortex, 3: lesion occupying 75% or more of the cortex. When the extent of the lesion was regarded to be between two grades, 0.5 was added to the lower score. These three scores were summed for each patient and expressed as his or her total pathological score.

**Cell culture**

CNT cells were seeded into a culture insert bottomed with a 0.4-μm pored semipermeable membrane coated with type I collagen (Sigma-Aldrich) using a 12-well two-chamber plate. Cells were grown to confluency under the standard culture condition (10% fetal calf serum and 21% O_2_), and the culture condition was kept as it was or changed as follows: A, the medium was changed to that containing 0.5% fetal calf serum; B, O_2_ concentration around the cell dishes was reduced to 15%; or C, A and B were combined. Then, the cultures were continued for 3 days, and cells were used for viability assessment (described below) or harvested to be subjected to Western blot analyses. In some experiments, the culture condition was restored to the standard, and the cultures were continued for another 2 days, then cells were harvested for Western analyses. For experiment B, the 12-well plate was put in a plastic container (AnaeroPack Box Jar, Mitsubishi Gas Chemical Company, Tokyo, Japan), together with an oxygen absorber–CO_2_ generator (AnaeroPack-CO_2_, Mitsubishi Gas Chemical Company), and was sealed off, according to the manufacturer’s instructions. The oxygen concentration was confirmed with an oximeter (OXY-1; Ichinen Jikco, Tokyo, Japan).

Cell viability and apoptosis was assessed using pSIVA Real-Time Apoptosis Fluorescent Microscopy Kit (Bio-Rad, Hercules, California, USA) according to the manufacturer’s instructions. Briefly, the culture medium was changed to the fresh one supplemented with 2 μL/mL of 1 M CaCl_2_ (final concentration: 2 mM Ca^2+^), and then the pSIVA-IANBD and PI staining solution were added to the medium at concentrations of 10 μL/mL and 5 μL/mL, respectively. The cells were observed alive under a confocal laser scanning microscope (C2+; Nikon, Tokyo, Japan) equipped with a CO_2_ chamber unit for cell culture. Then, the cells were fixed with formalin, and were stained with 4',6-diamidino-2-phenylindole (DAPI; Sigma-Aldrich) to visualize the nuclei and to assess cell confluency. Experiments were independently repeated five times with similar results.

**Western blot analysis**

Cells were harvested by centrifugation and the cell pellets were lysed in a buffer containing 50 mM Tris-HCl (pH 8.0), 150 mM NaCl, 1% Triton X-100 and 1 mM phenylmethylsulfonyl fluoride and were subjected to Western blot analyses as described in our previous report (Koma et al., 2008). Urine samples were directly separated on SDS-PAGE gels. Immunoreactive band intensities were quantified using ImageJ software (National Institutes of Health, Bethesda, MD, USA), as described previously (Mimae et al., 2012).

**Statistical analysis**

Associations between CADM1 concentrations and renal diseases were analyzed using the Steel–Dwass test. Correlations in ELISA standard curves, among renal parameters, and between CADM1 concentrations and Western blot intensities were analyzed using Pearson’s product-moment correlation tests. Changes in the correlation between eGFR and pathological scores were investigated with respect to the lower bound of urinary CADM1 concentration by using the cor.test function of the R programming language (https://www.r-project.org/). Ratios of Western blot intensities were analyzed using one-way ANOVA among all experimental groups, and the Bonferroni correction was applied to particular two groups. A *P*-value ≤ 0.05 was considered to indicate statistical significance.

**References**

Furuno, T., Ito, A., Koma, Y., Watabe, K., Yokozaki, H., Bienenstock, J., et al. (2005). The spermatogenic Ig superfamily/synaptic cell adhesion molecule mast-cell adhesion molecule promotes interaction with nerves. J Immunol 174(11), 6934-6942.

Kato, T., Hagiyama, M., Takashima, Y., Yoneshige, A., and Ito, A. (2018). Cell adhesion molecule-1 shedding induces apoptosis of renal epithelial cells and exacerbates human nephropathies. Am J Physiol Renal Physiol 314(3), F388-F398. doi: 10.1152/ajprenal.00385.2017.

Kato, T., Kurosawa, T.M., and Taketo, M.M. (2009). Proteinuria-induced chronic kidney disease in the ICGN/Oa mice with a mutation of Tensin2 gene. Ren Fail 31(3), 229-238. doi: 10.1080/08860220802669834.

Koma, Y., Furuno, T., Hagiyama, M., Hamaguchi, K., Nakanishi, M., Masuda, M., et al. (2008). Cell adhesion molecule 1 is a novel pancreatic-islet cell adhesion molecule that mediates nerve-islet cell interactions. Gastroenterology 134(5), 1544-1554. doi: 10.1053/j.gastro.2008.01.081.

Koma, Y., Ito, A., Wakayama, T., Watabe, K., Okada, M., Tsubota, N., et al. (2004). Cloning of a soluble isoform of the SgIGSF adhesion molecule that binds the extracellular domain of the membrane-bound isoform. Oncogene 23(33), 5687-5692. doi: 10.1038/sj.onc.1207761.

Mimae, T., Okada, M., Hagiyama, M., Miyata, Y., Tsutani, Y., Inoue, T., et al. (2012). Upregulation of notch2 and six1 is associated with progression of early-stage lung adenocarcinoma and a more aggressive phenotype at advanced stages. Clin Cancer Res 18(4), 945-955. doi: 10.1158/1078-0432.CCR-11-1946.

**Supplementary Figure Legends**

**Supplementary Figure S1 | Correlation between urinary CADM1 concentration and pathological scores.** Scatter plots of the patients whose CADM1 concentrations are larger than 362 (left) and 1000 (right) pg/ml. Approximate lines (dotted line), Pearson’s *R^2^* and *P*-values are shown.

**Supplementary Figure S2 | Correlation among urinary biomarkers, pathological scores, and eGFR.** (A) Urinary CADM1 concentration vs β2-microglobulin (left) or NAG (right). (B) eGFR vs total pathological score in various subsets of patients; β2-microglobulin (left) or NAG (right) are used as a cutoff to select patients, as indicated. (C) β2-microglobulin vs NAG. Approximate lines (dotted line), Pearson’s *R^2^* and *P*-values are shown. NAG, N-acetyl-β-D-glucosaminidase.

**Supplementary** **Figure S3 | Culture of CNT renal tubular cells.** CNT cells were cultured for 3 days under the normal (FBS 10%, O_2_ 21%; left) or ischemia-like (FBS 0.5%, O_2_ 15%; right) condition, and were then labeled with pSIVA and propidium iodide, as shown in Figure 5c of the main text. After fixed with formalin, cells were stained with DAPI to visualize the nuclei. Note that both cultures are confluent. Bar = 50 μm.
